# Dermal and Ophthalmic Findings in Pseudohypoaldosteronism

**DOI:** 10.4274/jcrpe.1740

**Published:** 2015-06-03

**Authors:** Sabriye Korkut, Emir Gökalp, Ahmet Özdemir, Selim Kurtoğlu, Şafak Demirtaş, Ülkü Gül, Osman Baştuğ

**Affiliations:** 1 Erciyes University Faculty of Medicine, Department of Neonatology, Kayseri, Turkey; 2 Erciyes University Faculty of Medicine, Department of Pediatrics, Kayseri, Turkey; 3 Erciyes University Faculty of Medicine, Department of Pediatric Endocrinology, Kayseri, Turkey

**Keywords:** Pseudohypoaldosteronism, newborn, Skin, dermal, ophthalmic, Eye

## Abstract

Pseudohypoaldosteronism (PHA) is defined as a state of resistance to aldosterone, a hormone crucial for electrolyte equilibrium. The genetically transmitted type of PHA is primary hypoaldosteronism. Secondary hypoaldosteronism develops as a result of hydronephrosis or hydroureter. PHA patients suffer from severe hyponatremia and a severe clinical condition due to severe loss of salt can be encountered in the neonatal period. Dermal findings in the form of miliaria rubra can also develop in these patients. With the loss of salt, abnormal accumulation of sebum in the eye due to a defect in the sodium channels can also occur. In this paper, a case of PHA in a newborn showing typical dermatological and ophthalmological findings is presented.

## INTRODUCTION

Aldosterone is a hormone which plays an important role in electrolyte balance in the human body. A state of resistance to aldosterone is known as pseudohypoaldosteronism (PHA). PHA types with genetic transmission are called primary PHA, while those resulting from hydronephrosis and other urogenital problems are called secondary PHA (1,2,3). Although a state of salt depletion characterized by hyponatremia, hyperpotassemia and metabolic acidosis may occur due to other causes, detection of a high serum aldosterone along with these findings usually leads to a diagnosis of PHA (1,4,5,6). Primary PHA type 1 is the most frequently encountered form. Inheritance of type 1 PHA cases can be autosomal recessive or autosomal dominant (6). The autosomal dominant form, with aldosterone receptor resistance restricted to the kidneys only and generally resolving in early childhood, is clinically milder. The problems encountered in cases with autosomal recessive forms of PHA stem from the mutations in the genes related with the subunit of epithelium sodium channels. Known as systemic PHA 1, this form involves, in addition to the kidneys, the sweat glands, the distal colon, the lungs, the reproductive organs and the salivary glands and its clinical figure follows a more acute course (7). These patients may present in the neonatal period in a severe state due to grave loss of salt and cardiac arrhythmia (8). They may also present with respiratory distress, pulmonary infection and cystic fibrosis-like symptoms (9). Dermal and ophthalmic findings may be present in PHA cases and these findings may be of great importance for early diagnosis (10,11,12,13,**14**). During the attacks of salt depletion, miliaria rubra may be observed in the skin resulting from the accumulation and excretion of sodium in sweat glands (**10**,**11**). Abnormal accumulation of sebum in the eyes is quite typical and may also occur in secondary infections (**12**,**13**,**14**). In this paper, we present a patient who was hospitalized with miliaria and specific ophthalmic findings in the neonatal period and a review of the relevant literature.

## CASE REPORT

Our patient was a female baby referred to our clinic on postpartum 7th day with a skin rash and lacrimation. The infant was reported to be delivered by caesarean section at a gestational age of 39 weeks. Weight at birth was 3050 g. The baby had been given to her mother immediately after birth, she had been discharged on postnatal day 2, only to be returned the following day with erythema and lacrimation. It was also learned that, despite a treatment consisting of an ointment for her skin and application of an antibiotic containing eye drop, her condition had not been resolved. The mother’s history revealed that throughout her pregnancy, which had a normal course, she had attended all her follow-up visits, that she and her husband were cousins and their first child had died of an infection on the 10th postpartum day.

**Figure 1:**
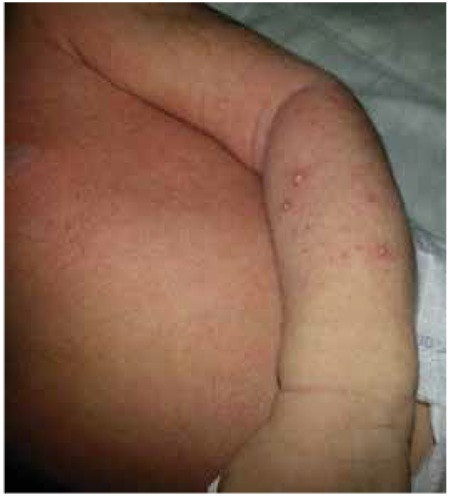
The appearance of the miliaria rubra on the trunk and arm.

**Figure 2:**
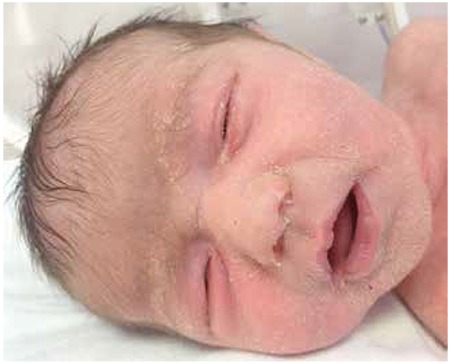
The appearance of salt crystals at the base of the follicles of the face.

**Figure 3:**
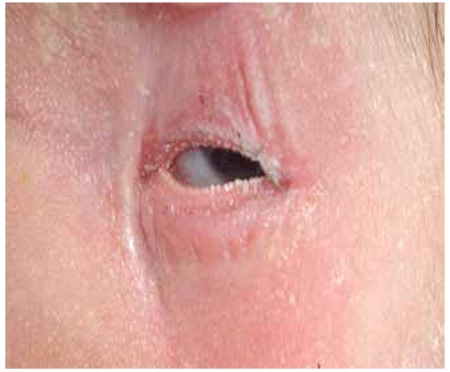
Presence of a white opaque discharge with an appearance of a row of teeth at the bottom of the eyelashes.

At admission, the infant’s body weight was 2740 g (10th-25th percentiles). Her length was 52 cm (50th-75th percentiles) and her head circumference was 35 cm (50th percentile). The general condition of the patient appeared to be good and she was active. Miliaria rubra were noted on her face, her arms and her chest (Figure 1). Presence of salt crystals could be detected at the base of the follicles of the face (Figure 2). There was a white opaque discharge at the bottom of the eyelashes and macerations around the eye were noted (Figure 3). The remaining systemic examination findings were normal. Laboratory assessment revealed a hemoglobin level of 19.5 g/dL, WBC 18870/mm3, a thrombocyte count of 412000/mm3, a hematocrit level of 59%. C-reactive protein level was 3.44 mg/L, blood glucose 82 mg/dL, blood urea nitrogen 22 mg/dL, creatinine 0.4 mg/dL, sodium 129 mEq/L, potassium 6.5 mEq/L, calcium 10.8 gm/dL and phosphorus was 6.4 gm/dL.

The patient was treated with appropriate fluid replacement. However, the following day her plasma sodium was 129 mEq/L, potassium was 8.1 mEq/L and urine sodium was 31 mEq/L.

Because of polyuria, her maintenance fluid requirement was calculated taking into consideration the insensible water losses and amount of urine. The patient was monitored and given treatment for hyperpotassemia and sodium deficiency. The etiology of the adrenal insufficiency was investigated. At ultrasonic imaging, the length and thickness of the adrenal cortex was reported as 16.0x4 mm on the right and 16.5x3.5 mm on the left. The glands were cribriform in appearance, which is in keeping with findings in congenital adrenal hyperplasia (CAH). With a provisional diagnosis of adrenal insufficiency, the patient was started on hydrocortisone therapy. In blood samples taken before hydrocortisone therapy, plasma adrenocorticotropic hormone level was 5.73 pg/mL, cortisol level 31 ug/dL and 17-hydroxyprogesterone level was 1.87 ng/mL. These findings led us to reconsider our initial diagnosis of CAH. A tentative diagnosis of hypoaldosteronism was considered. The patient’s plasma aldosterone was found to be over 6000 pg/mL (normal ranges 50-1750 pg/mL). Plasma renin activity was 100 ng/mL (normal ranges 2-5 ng/mL) and saliva sodium level was 152 mEq/L (normal ranges 33.1±13.4 meq/L) (15). These findings were considered to be consistent with a diagnosis of systemic PHA. The patient is still being monitored in our unit and is continuing to be treated with 0.3 mg/day of fludrocortisone and also receiving treatment for sodium deficit and hyperpotassemia (glucose buffered insulin, nebulized salbutamol, calcium gluconate and oral kayexalate).

## DISCUSSION

Critical conditions due to salt loss in the neonatal period arise as a result of problems with adrenal hyperplasia which cause salt loss, such as CAH, or of problems with aldosterone secretion (16). Aldosterone deficiency in cases of CAH is observed in the absence of aldosterone synthase. The presence of aldosterone in high levels despite the crisis of severe salt loss leads to a diagnosis of PHA. The first newborn case was published in 1958 (17).

Two points which cause errors in diagnostic studies related to PHA must be kept in mind. The first of these is that the aldosterone level, which is very high in PHA, may turn out to be low in the first phase as a result of the hook effect caused by the high level of aldosterone (18). The second is that aldosterone levels may not be high in cases of severe sepsis and CAH, as a result of the interference of other hormones and their precursors in serum samples not subjected to extraction and purification (19). Therefore, it is necessary to quantify the 17-hydroxyprogesterone level of the patient, to obtain adrenal ultrasonography results and to do a genetic study before reaching a diagnosis of PHA and discarding a diagnosis of CAH or other pathology relating to the adrenal glands. Congenital hydronephrosis and hydroureter cases can also be detected early by renal US. In patients with PHA, it is also essential to quantify the sodium and potassium levels in the serum, urine, sweat glands and saliva for the differential diagnosis of the subtypes of PHA. The fact that the sodium level in saliva was found to be high in our case is a finding which supports a diagnosis of PHA type 1 in autosomal recessive systemic form (1,4,5,6). In the neonatal period, apart from hyponatremia and hyperpotassemia in the clinical crisis resulting from severe salt loss due to PHA, thrombocytosis is also striking (11). Indeed, our patient had thrombocytosis.

Several diseases may cause a salt-wasting state during infancy, notably severe diarrhea, endocrine abnormalities such as CAH, renal disease and cystic fibrosis. However, none of these conditions are associated with a specific eye abnormality, whereas the detection of dermal and ophthalmic signs in PHA cases may be of great importance for early diagnosis (12). Also in PHA cases, an elevated concentration of salt in the sweat causes cutaneous lesions similar to those which appear in miliaria rubra (10,11). A hypothesis for the cause of skin rash is that secretion of sweat with an elevated sodium chloride concentration directly damages the eccrine ducts. This hypothesis is supported by the fact that this eruption appears during a salt-depletion crisis when the sodium chloride concentration in the sweat is highest. The elevated sodium chloride in the sweat also contributes to formation of inflammatory lesions around the eccrine glands (10). Another typical sign in patients is the presence of ophthalmic problems. The function of the meibomian glands arranged in the ophthalmic tarsal plaque is to produce lipid compounds to prevent drying via evaporation (12). Since the epithelial sodium channels are inactive in PHA cases, formations looking like rows of teeth can appear owing to the accumulation of salt and sebum in the meibomian glands (12,14). Our patient initially presented with skin rash and discharge from the eyes and was followed with a diagnosis of conjunctivitis and infectious dermatitis. It is difficult to differentiate skin lesions in these patients from other nonspecific skin lesions, whereas ocular findings are very typical.

In summary, in this paper, reference was made to the dermal and ophthalmic findings observed in a case of autosomal recessive PHA type 1 hospitalized in the neonatal unit with severe loss of salt and the importance of these findings in providing diagnostic guidance was emphasized.
